# A qualitative study examining the validity and comprehensibility of physical activity items: developed and tested in children with juvenile idiopathic arthritis

**DOI:** 10.1186/s12969-019-0317-6

**Published:** 2019-04-25

**Authors:** August Flodén, Eva W. Broström, Johan von Heideken, Sara Rostlund, Rikard Nilsson, Kristina Löwing, Maura D. Iversen

**Affiliations:** 1 Department of Physical Therapy, Stockholm South General Hospital, Stockholm, Sweden; 2 Department of Women’s and Children’s Health, Karolinska Institutet, Stockholm, Sweden; 3 Section of Clinical Sciences, Department of Medicine, Brigham & Women’s Hospital, Boston, MA USA; 4 Department of Physical Therapy, Movement and Rehabilitation Sciences, Northeastern University, 360 Huntington Avenue 120 E Beharkis Health Sciences Building, Boston, MA 02115 USA

**Keywords:** Juvenile idiopathic arthritis, Self-reported physical activity, Qualitative study

## Abstract

**Background:**

Not all physical activity (PA) questionnaires (PAQ) gather information regarding PA intensity, duration, and modes and only a few were developed specifically for children. We assessed children’s comprehensibility of items derived from two published PAQs used in children along with three items designed to ascertain PA intensity in order to assess comprehensibility of items and identify response errors. We modified items to create a new PAQ for children (**ASCeND**). We hypothesized that children would have comprehension difficulties with some original PAQ items and that **ASCeND** would be easier to comprehend, and would improve recall and reporting of PA.

**Methods:**

For this qualitative study, we recruited 30 Swedish children [ages 10–16 years; mean age = 13.0 (SD = 1.8)]; median disease activity score = 4.5 (IQR 2.2–9.0); median disease duration = 5.0 (IQR 2.6–10.8) with juvenile idiopathic arthritis (JIA) from a children’s hospital-based rheumatology clinic. We conducted cognitive interviews to identify children’s comprehension of PAQ items. Interviews were audiotaped, transcribed, and independently analyzed. In phase one, 10 children were interviewed and items modified based on feedback. In phase two, an additional 20 children were interviewed to gather more feedback and further refine the modified items, to create the **ASCeND.**

**Results:**

The median interview time was 41 min (IQR 36–56). In phase one, 219 comments were generated regarding directions for recording PA duration, and transportation use, walking, dancing, weight-bearing exercise and cardio fitness. Based on feedback we modified the survey layout, clarified directions and collapsed or defined items to reduce redundancy. In phase two, 95 comments were generated. Most comments related to aerobic fitness and strenuous PA. Children had difficulty recalling total walking and other activities per day. Children used the weather on a particular day, sports practice, or gym schedules to recall time performing activities. The most comments regarding comprehension were generated about the 3-item PA intensity survey, suggesting children had problems responding to intensity items.

**Conclusions:**

The newer layout facilitated recall of directions or efficiency in answering items. The 3-item intensity survey was difficult to answer. Sports-specific items helped children more accurately recall the amount of daily PA. The ASCeND appeared to be easy to answer and to comprehend.

**Electronic supplementary material:**

The online version of this article (10.1186/s12969-019-0317-6) contains supplementary material, which is available to authorized users.

## Background

Inadequate physical activity (PA) is increasingly common in younger people [1, 2] and especially among children with chronic disability [3–5]. Juvenile Idiopathic Arthritis (JIA) is a systemic autoimmune disease with a prevalence of 0.16% in the Swedish population in children up to the age of 16 years [6]. JIA is traditionally characterized by articular and extra-articular symptoms (ocular, cardiac, pulmonary and hematopoietic) that may impact physical and psychosocial function. PA levels in children with JIA are rarely studied but trends show these children demonstrate lower PA levels than their healthy peers [7–9]. These signs of lower PA levels are alarming as physical activity positively impacts health, social engagement and development, and reduces joint symptoms and stress [10]. Additionally, physical activity at a younger age influences later cardiovascular health as indicated by the fact that as children with JIA become adults, they demonstrate a higher prevalence of arterial calcification when compared with healthy peers [11]. At present, interventions to improve PA levels of these children reveal no clear effect on function during activities nor on lifestyle (habitual) PA [10].

Accurate assessment of PA is required to identify actual PA engagement and the impact of interventions at the individual or population level [12]. Self-report measurements are considered feasible for epidemiological studies as they are easy to administer, are low cost, have minimal participant burden and are generally well accepted [13]. However, self-report measures are influenced by recall and response bias and may not capture absolute levels of PA. Direct PA measures (i.e. calorimetry, motion sensors or direct observation) are often considered more capable of precisely estimating energy expenditure and remove the inherent issues of recall and response bias [14]. Despite the greater accuracy of direct measures, they are time and cost intensive, and rely on patient adherence (e.g. wearing a monitor or using a smartphone to collect data) rendering them less useful in epidemiologic settings. In children, adherence can be challenging, as many schools do not allow children to have devices on their person when participating in classes, sports, or other physical activities. As such, no single “gold standard” for assessing and validating PA in children exists today [15].

A number of PA questionnaires exist for children (see Additional file 1) [16–30]**.** Some assess PA during the school year, others assess PA during the school week, some assess modes of activities but not frequency, and others assess frequency and modes but over a 7 day week. Presently, no pediatric measurement exists that adequately captures habitual PA in all its dimensions (intensity, frequency, mode, and duration). Even though the study by Singh-Grewal et al. [31] demonstrated that PA intensity level did not change the outcomes of children with JIA, it is important to measure exercise intensity to determine whether PA levels of these children meet current PA Guidelines. As PA is believed to mediate disease activity and bodily function in JIA [10], there is a need for an accurate, cost-effective and feasible instrument to assess all aspect of PA in these children. This study aimed to evaluate the appropriateness, comprehensibility, and sources of response errors of items derived from two PA questionnaires (PAQ-A [19] and Active-Q [17]) and modified to include three items ascertaining PA intensity when administered to Swedish children with JIA aged 10 to 16 years. We hypothesized that children would have difficulty with the appropriateness and comprehensibility of some original PAQ items and that the new “Activity Scale for Children with Different Abilities”(**ASCeND**), would be easier to comprehend and would improve recall and description of total PA levels.

## Materials and methods

### Sampling method and recruitment

**Fig. 1:**
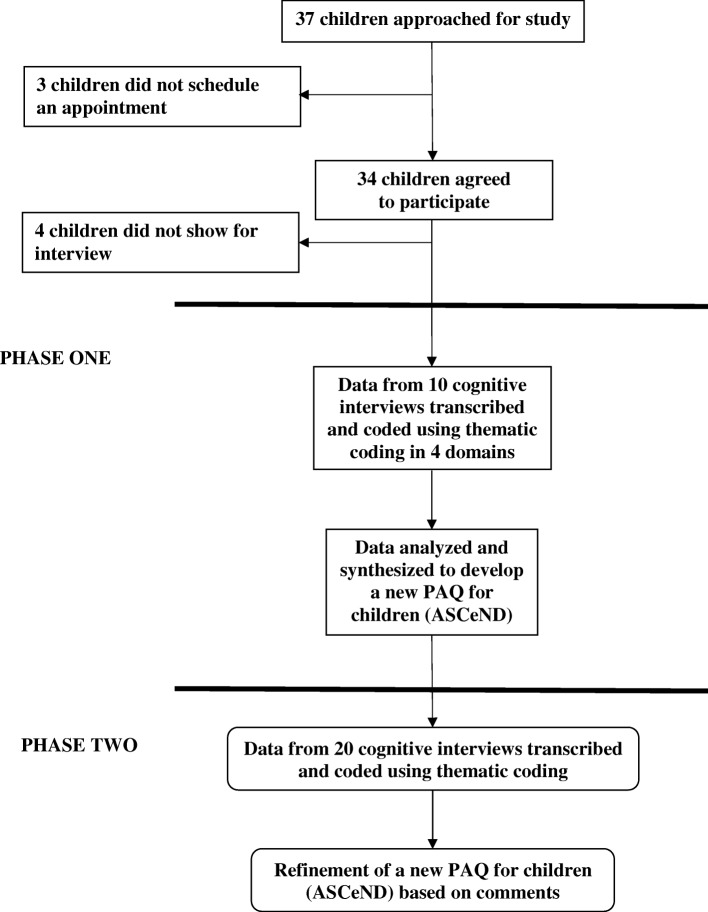
Flow diagram showing enrollment, lost to follow-up, drop outs and analyses of the transcripts

Institutional approval was obtained for this qualitative study. We recruited consecutive patients with a primary diagnosis of JIA, ages 10 to 16 years, who came to the rheumatology clinic who met our study criteria. This clinic was located in a high-volume, urban, tertiary-care pediatric medical center. We aimed to recruit 30 Swedish children as this sample size has been shown to be effective in studies of survey comprehensibility among children these ages [32–35]. Children provided assent and their parents provided informed consent. Children did not receive compensation for participation. To obtain equal representation by age and gender, children were purposefully sampled in blocks by age (10 to < 12, 12 to < 14, 14 to 16 years) and gender (Fig. 1). Baseline demographic data were collected from medical records and included type of JIA, medications, disease duration, and disease activity status, assessed by clinical Juvenile Arthritis Disease Activity Score (JADAS-71) [36]. JADAS-71 scores are interpreted as follows for oligoarthritis: ≤ 1 inactive disease; 1.1–2.0 for low disease activity; 2.1–4.2 moderate disease activity and > 4.2 high disease activity; and for polyarthritis: ≤ 1 inactive disease; 1.1–3.8 low disease activity; 3.9–10.5 moderate disease activity and > 10.5 high disease activity [37]. During interviews we also collected self-reported sports and play.

Cognitive interviewing, the gold standard in survey development and assessment, was used to ascertain children’s comprehension of items and directions, what they believed the items intended to ask, what processes they used to answer items, and whether they were able to find an answer that fit how they wished to express themselves when answering an item [32]. Children under 10 years of the age were not included due to their limited ability to use lexical comprehension when answering self-reported questionnaires [32]. During interviews, we used both concurrent “think aloud” and retrospective probing to ascertain comprehension of the measures. Research indicates such approaches when delivered in a standardized format by trained researchers, is an effective and unbiased method used to uncover thinking associated with responses to surveys [38].

Prior to the interviews, we extracted items from two previously published PAQs [17, 19]. Items extracted from these scales are similar in content, although the layouts, inclusion of intensity, and response sets vary. We included all activity specific items that were consistent across the PAQs as well as unique items from individual PAQs. We then modified the survey directions to ensure activity reporting was not limited to the school week/school year but to the past 7 days. In addition, we included 3 new items regarding amount of physical activity per week stratified by intensity level. Next, an independent person fluent in English and Swedish translated the survey into Swedish. A second person who was also fluent in both English and Swedish independently back translated the Swedish version of the survey to ensure accuracy in translation. Items were then reviewed for discrepancies and changes made based on consensus. We included the 3-item PA intensity survey as this format of ascertaining physical activity has been suggested as a more efficient way of gathering this data.

The study was divided into two phases. In phase one, based on the volume of feedback from cognitive interviews of the first 10 children enrolled (mean age = 12.7 years SD = 1.5) items were modified the survey. In the second phase, the next 20 children enrolled (mean age = 13.1 SD = 2.0) provided feedback on the modified survey to refine items and formatting and test the newly developed survey, the Physical Activity Scale for Children with Different Abilities (**ASCeND)**. There were no differences with respect to demographic features of participants in phase one and phase two (Table 1).

**Table 1:** Characteristics of children participating in cognitive interviews to evaluate the physical activity outcome measures (*n* = 30)

Variable	Phase 1 (*n* = 10)	Phase 2 (*n* = 20)	*p*-value^*^
Mean age in years on date of interview (SD)	12.7 (1.5)	13.1 (2.0)	1.00
Female	5 (50%)	10 (50%)	NS
Type of JIA			
Polyarthritis	3	4	
Oligoarthritis	3	11	
Monoarthritis	0	1	
Psoriatic arthritis	2	1	
Systemic arthritis	1	2	
Spondyloarthritis/enthesitis	1	1	
Mean duration of JIA, years (SD)	7.2 (4.1)	6.2 (4.7)	0.53
Median disease Activity, JADAS71	4.8 (0.4–9.4)^b,a^	4.5 (2.7–9.0)^c,a^	0.70
Inactive disease activity	3	3	
Low disease activity	0	1	
Moderate disease activity	3	2	
High disease activity	3	12	
Number of active joints per child	2 (0–4)^b,a^	1 (0.25–2)^d,a^	0.76
Number of active joints in the lower extremity per child	2 (0–2)^b,a^	1 (0–2)^d,a^	0.80
Number of joints with restricted range-of-motion	0 (0–0)^b,a^	0 (0–0)^d,a^	0.45
Number of joints with restricted range-of-motion in lower extremity per child	0 (0–0)^b,a^	0 (0–0)^d,a^	0.80
Medications			
Steroids	0	1	
Biologic and Steroids	2	0	
Biologic and DMARDs	6	1	
Biologic	1	4	
NSAIDs	1	5	
DMARDs	0	6	
No medication	0	3	
Mean KOOS Sports Score (SD)	0.6 (0.7)^b^	1.0 (0.7)^c^	0.69

*JADAS71* Juvenile Arthritis Disease Activity Score

Lower extremity joints includes all joints in lower limb, excluding sacroiliac joints

^*^Phase 1 vs. Phase 2, Independent-Samples Median Test for testing the homogeneity between the groups. ^a^median and Interquartile range; ^b^missing 1; ^c^missing 2; ^d^missing 4

### Standardized training of interviewers

An experienced behavioral scientist (MDI), who has extensive experience in the application of cognitive interviews for survey development, created the interview protocol and the specific and general verbal probes [32, 33]. The behavioral scientist then trained the interviewer in cognitive interviewing procedures and techniques (AF), until he was deemed proficient in the interview process. This protocol has been utilized in multiple previous studies [33, 34, 39, 40].

### Interview procedure

Parents were allowed to remain present during the interview, but were instructed not to respond for the child. The interviewer explained the purpose of the interview and asked a few basic questions about the primary diagnosis and current PA level: (1) “How long have you had your diagnosis?” (2) “What typical physical activities do you do in your spare time?” (3) “Where do you live”?

Following these introductory questions, each child was given the PAQ and asked to read the directions aloud before answering the survey items. The child was then asked to describe the directions in his or her own words. The interviewer observed the child while the child completed the surveys and recorded whether the child hesitated or demonstrated signs of difficulty completing the items. Next, using standardized and general verbal probes, the interviewer discussed the child’s interpretation of the survey’s directions, items, and item responses (Table 2) [38, 41–43]. All interviews were audiotaped, recorded, and transcribed. Transcripts were then examined and coded based on four content areas: (1) item comprehension (language/jargon/lexical), (2) information retrieval and recall strategies, (3) decision-making processes, and (4) response mapping to identify how the child’s answers aligned with the response sets provided [32, 33].

**Table 2:** Specific and general probes used during cognitive Interviewing of physical activity questions in children with JIA (phase one and two)

Current ASCeND items	Specific probes	General probes
Walking	What do you think we mean by “walking”?	How did you choose your response?
Swimming	How do you remember how much time you spent swimming on the day you wrote down?	How did you arrive at your answer for each item? “Are the answers easy to understand?” What activities would you like to include that are not included in the survey?
Survey Directions	What does “physical activities” mean to you?	“How would you change the directions to make them clearer?” “Are the answers easy to understand?”
Performing a physical activity that requires you to breathe hard	Please explain what this question is asking you in your own words.	How would you describe item X to your friends? “What information in the possible answers helped you make your choice?”
Sitting	How certain are you of your answers?	How did you select your answer? What does the sentence “Past 7 days” mean to you?
Ball sports	You stopped for a moment on ball-sports, can you tell me your line of thinking?	“Do you have any suggestions for us regarding the directions on the form or items?”

### Analysis

Transcripts were read, coded, analyzed independently and synthesized using thematic coding by four research team members (AF, JvH, RN, MDI). The team consisted of behavioral scientist (MDI) and a pediatric orthopedic surgeon (JvH) both experienced in cognitive interviewing and survey development [32–35], and two physical therapists (AF and RN) who had 3.5 and 2.5 years of experience in pediatrics and arthritis, respectively, to identify problem detection, which included counts of issues arising when the children completed the questionnaires [38, 44]. Problematic items were then sorted to identify the specific source of error (comprehension, mapping, response or stem format) and shared with the team members. Additionally, the team reviewed children’s comments regarding general and specific directions, survey format, and difficult words using a normative group process. The team came to a consensus on word and format changes to improve survey readability. Based on the data gathered in the cognitive interviews and from specific recommendations by children for rephrasing items, we created a new preliminary survey. The study team reviewed the survey modifications using a normative process and came to a consensus about the changes. Thus, the survey format was revised to enable children to see the directions for the section along with a larger list of PA items.

Once modifications were made to the **ASCeND**, we tested the survey among the next consecutively recruited 20 children (50% female, mean disease duration = 6.2 years, SD = 4.7) using the same cognitive interviewing and analytic techniques to assess comprehension of the new measure.

#### Statistical analysis

We used the statistical program SPSS for Mac 24.0 (SPSS Inc., Chicago, IL, www.spss.com) to calculate descriptive statistics and perform inferential tests. Continuous data are presented as means (min-max); categorical data, as frequencies and percentages. We examined demographic data to determine whether significant differences existed between the subjects in Phase One and Phase Two, using a Fisher’s exact test, t-tests and Mann-Whitney U tests, as appropriate. We tested for differences in the number of comments mentioned between genders using a Mann Whitney U test and a Kruskal-Wallis test for differences in the number of comments between the three age groups. Additionally, as a preliminary assessment of concurrent validity of PAQ items, we compared the total minutes of self-reported PA from activity-based items (excluding sedentary activity) with the total minutes of PA reported using the 3 PA intensity items among subjects in Phase 2, using spearman rank correlation coefficients.

## Results

The mean age of the 30 children was 13.0 years (SD = 1.8) and median disease duration was 5.0 years (IQR 2.6–10.8)]. Of the 30 children, 6 (20%) had inactive disease and 15 (50%) had high disease activity. (Table 1) The median interview length across both study phases was 41 min (IQR = 36–56). Within phase one, the median interview time was 45 min (IQR = 37–57) and within phase two, the median interview length was 39 min (IQR = 34–53). Children reported engaging the most in soccer, videogames, and dancing.

### Phase one

In phase one, the 10 children made 219 comments about the survey. Transcript data were sorted into the following categories: comprehension of language (e.g. reading level/phrasing/lexical) and medical jargon, item format (content of directions and timeframe, double-barreled items (e.g. asking about more than one symptom in a single item), response set format (terminology), and response mapping (responses available but not considered by the children to be suitable). The most problematic items were: ascertaining PA intensity level (total 32 comments), followed by amount of time performing chores, time spent sitting or lying down, directions for recording amount of time spent engaging in specific activities, time spent watching shows on the computer, phone or other device, and items that asked children to report the amount of time performing an activity for travel or for fun (e.g. walking dog versus walking to school). All children found the survey layout interfered with their ability to recall the directions for the activity specific items.

Items requesting the amount of time traveling via various vehicles were collapsed into a general category of traveling by vehicle, regardless of the vehicle type. Suggestions for wording directions were incorporated to clarify what was asked of children. We also provided examples of cardio exercise and weight bearing exercises and removed the general category general exercise.

Item ordering was modified so that general PA items (aerobic fitness, sitting and reading) were placed after specific activities. The change in item ordering addressed the issue of double counting time when reporting on specific and general activities. For example, when asked to explain his answers for cardio-related activities, one child responded, ***“Mhm, on Mondays its... When we play football during recess, then I do 20–30 min; the same on Wednesday and on Tuesday we had football-practice as well.”*** and then answered the same time-quantities for ball-sports and cardio-activities.

"We modified the phone item", allowing children to decide whether their time on the phone accounts for texting, reading, playing games, or watching shows. Children found everyday tasks to be the most problematic when reporting time. However, activities that were hobbies or joyful seemed easier for them to recount. For example, one child stated, ***“[…] if I ride (a horse) at the stable then maybe I ride for 45-50 minutes, and I know that because I’ve been riding for very long. I know about how much I ride, [….].”****.* Whereas another child said, ***“[…] When you go shopping on your shopping-spree, it is easy to keep track of time. I can be in a store for an hour or so. So it’s not hard to be in a store for an hour […]”***.

### Phase two: feedback and refinement of ASCeND

Using an iterative design approach, this revised survey was tested among the remaining 20 consecutively enrolled children. In total 95 comments were generated during these interviews. Table 3 illustrates the comments per item by category (lexical, response format etc.). Issues related primarily to response format, stem format, and comprehension. Items that generated the most comments (5–6 comments each) were aerobics or cardio exercise and the general time spent performing various intensities of PA, (e.g. mild, moderate, and strenuous). To enhance clarity, directions were added across the top of each page of the survey versus only at the beginning of each section. We also added directions to the section on sedentary activities (e.g. sitting, sleeping and lying down) to emphasize the fact that the amount of time spent doing these activities should be tallied regardless of what children were doing in these positions. Of all the activities, squash generated the most confusion, as several children found the word foreign and did not understand its meaning. Squash was therefore removed from the activity list as it is not commonly played in Sweden and due to the fact that the “other racquet-sports” item would likely cover this specific activity. Next, we refined the wording on the three intensity items.

**Table 3:** Summary of issues arising from cognitive interviews with children with JIA regarding physical activity items (Phase 2) (*n* = 20)

	Stem Format general	Stem Comprehension-Terminology	Stem Format- Lexical^^	Stem Format- Double- barreled^	Response Format	Response Double Barreled	Response Mapping
Number of Children Reporting These Issues							
Over the past 7 days, which of the following activities have you done? Record the amount of days per week and for how long.							
Overall number of comments	22	23	6	3	39		2
Directions Part 1	6						
Walking	1				1		
Cycling							
By moped or scooter			1				
Riding in a vehicle (car, train, bus, subway or ferry)	1	1	1	1	1		
Sat down and read a book, writing or sewing					1		
Watch movies, series or a show							
Played computer or TV-games					1		
Played a music instrument, computer- or TV-game where you are standing up or moving					1		
Doing household chores, cleaning, laundry, taking out the trash etc.			1		1		
Shopping or other errands		1			1		1
Aerobics or cardio fitness class (e.g. zumba, core, body pump)	6	3	1		1		
Weight lifting		1			1		
Jogging, running or orienteering	1				1		
Athletics (e.g. high jump, long jump or three-step)					1		
Swimming					1		
Ball sports (e.g. soccer, basketball, volley ball or floor-ball)	1				1		
Golf					1		
Horseback riding		1		1	1		
Dance					1		
Dance-class or competitive dancing	1				1		
Skating, ice-hockey					1		
Skiing (downhill or cross-country)	1				1		
Martial arts (e.g. judo or karate)					1		
Boxing or wrestling					1		
Tennis, badminton or table tennis					1		
Squash	1	4			1		
Sailing, surfing, canoeing or rowing		1			1		
Motor sports (e.g. motocross)			1		1		
Rock climbing					1		
Yoga, tai-chi or pilates					1		
Mountain-bike or biking in demanding terrain					1		
Directions for intensity	1	1	1	1	1		
Heavily exhausting physical activity		3			2		
Moderately exhausting physical activity		2			2		
Not exhausting physical activity		1			2		
Directions for sleep/sit /lie	1	1			1		
How much have you slept (both at day and night)?					1		
How much have you been laying down? (not sleeping)					1		
How much have you been sitting?		1			1		
Compared to others your age, how would you describe your activity level?	1	2					1

^double-barreled items refer to more than one activity or symptoms in the stem or response

^^lexical refers to words or vocabulary of a language used

Fitness and aerobic activities remained problematic throughout phase two. For example, one child commented, **“I counted that time in biking as it is fitness”** and **“Aah! I counted that when I was working out [...][...] handball was here”**. The aerobic item was then moved from beginning of sports-related questions to the end of the survey to reduce duplication in reporting time. Finally, we made sure to move all sports-specific activities to the beginning of the survey to reduce duplication in reporting total PA. For final versions of the ASCeND survey in English and Swedish please refer to Additional files 2 and 3.

We examined the data to determine there were any differences in the total number of comments generated by age or by gender. Based on the results, there were no differences by gender and age group regarding the total number of comments generated during phase two of the interviews (median number of comments for boys and girls was 2; *p* = 0.85 and number of comments by age group was (ages 10 to < 12 median = 4 (IQR = 1–19), 12 to < 14 median = 1(IQR = 0–4), ages 14 to 16 years median = 2 (IQR = 1–3); *p* = 0.37). When assessing concurrent validity, we found a moderate but significant correlation between self-reported similar amounts of PA using the activity based PA items and the PA intensity items in the ASCeND (r = 0.43; *p* = 0.048).

## Discussion

This study aimed to evaluate the appropriateness, comprehensibility, and sources of response errors of items obtained from two PAQs [17, 19] as well as 3 items inquiring about intensity of PA of when administered to Swedish children with JIA aged 10 to 16 years. We confirmed our hypotheses that children would have difficulty with the appropriateness and comprehensibility of some original PAQ items and that the new PAQ, the Physical Activity Scale for Children (**ASCeND**), would be more comprehensible and would improve both recall and description of total PA levels.

Physical activity engagement includes the frequency, intensity, mode, and duration of activities. Outcomes of PA include metabolic equivalents (defined as the amount of oxygen consumed while sitting at rest and is equal to 3.5 ml O_2_ per kg body weight x minutes), amount of time engaged in PA, and intensity of PA. Physical activity can be measured using tracking devices such as pedometers or accelerometers or by PAQs. However, some PAQs ascertain rate of exertion instead of frequency or duration of activities (e.g. Holtebekk [45]). Thus, variability in the measurement of self-reported PA influences concurrent validity testing [3].

The ability of children to recall PA participation is debated [46–48]. In a recent study by Ambrust et al. [48] children with JIA ages 8 to 13 years were asked to record their PA using activity diaries while concurrently wearing an accelerometer. The authors found the validity of activity diaries to be low to moderately associated with accelerometer measures of PA; with activity diaries overestimating PA levels. A review by White et al. found that the most reliable, valid, and common recall period for self-report of PA in children with disabilities is 7 days [47]. Many PAQs use a 7-day recall period but collect PA in different ways. Some collect data on PA engagement during a typical week over the past year (ACTIVE-Q [17]), ask how often you engage in 60 min of PA over a typical or usual week (PACE [28]), collect data on activities during school lunch hour, the frequency of sports before and after school, activity engagement by normal school week and weekend (ASAQ [24]), and frequency of engagement in sports on evenings and weekends (e.g. PAQ-A & PAQ-C [19, 20]). The PAQ-C also includes frequency of PA engagement in recess and physical education class, as these are typically part of a younger child’s day in school. Our survey, **AScEND,** uses a 7-day recall period as this appears to be the best format for recall of PA but stipulates that children should report PA over the past 7 days versus week. We believe this is an important distinction to make as children use different anchors for a week (school week- begins on Monday or regular week which starts on Sunday (KOOS-child [33] and pedi-IKDC [39]).

Previous studies have also demonstrated that children’s ability to recall PA activities are less specific than adults, perhaps because some of their activity behaviors are less structured (e.g. [play) and/or because the variability in their daily activities is so high [49–51]. Some PAQs ask children to write in what activities they engage in and record the time spent on these activities. (7 day PAR [21]). Other PAQs provide a long list of specific sports and leisure activities and ask children to report the frequency they engage in these activities. (3DPAR [16], ACTIVE-Q [17], PAQ-A [19], PAQ-C [20], CLASS [18], AQAA [23], ASAQ [24]). Each of these approaches has strengths and limitations. The **ASCeND** provides a list of specific activities and asks children to report the frequency of PA engagement over the past 7 days. The modified format of **ASCeND** helps children report many activities on a page while reducing response burden. The horizontal grid format for each activity was also easier for children to align the response sets with corresponding items.

Our results suggest the use of activity specific items were easier for children to answer than the 3 PA intensity items, as noted by the markedly fewer comments on activity specific items compared to intensity items. We can only speculate about the reasons for the differences in amount of PA recorded using activity-specific items versus the 3-item PAQ intensity. We believe children require prompts in order to recall the activities they engage in over time as their activity levels are highly variable [52].

Current recommendations for PA in children emphasize the need to include multiple modes of exercise (strengthening, aerobic, and flexibility) and a total of 60 min of PA per day. Different modes of exercise provide different physiologic benefits, as does the intensity of exercise. Differences in exercise intensity also yield different health benefits (eg. cardiovascular versus strengthening). Thus, understanding the intensity of bicycling would indicate whether the physiologic benefit is strength or aerobic fitness. The use of sports-specific items then may be more beneficial when assessing PA in children.

In follow-up discussions, the study participants were asked if they felt any question was missing or redundant. The first version of the ASCeND used questions based on “the purpose” of the activity (e.g. walking at work, walking for leisure per published measures) and listed activities without a proposed purpose (e.g. walking), as two separate activities. Several adult PA-instruments use this format, such as the IPAQ [53]. Children reported issues of double-barreling their responses during the first phase of the testing. In phase 2, we did not separate activities based on their purpose but rather listed them based on the type of activity. No child expressed difficulty responding to these items in phase-2 testing.

Physical activity in JIA-populations has been studied previously [5, 7, 9, 54–59]. However, the instruments used in these studies have not been specifically developed and tested among children with disabilities. Research indicates that pain can mediate the ability to recall activities [60] and the prevalence of pain is a major differentiator between children with JIA and healthy peers [61–63]. The testing of comprehensibility and construct validity are key to improving questionnaires [38]. We based on the A**S**CeND on well-established items from current questionnaires and ascertained understanding of these items among children with JIA.

### Strengths and limitations

This study has several strengths. Children were recruited from a pediatric rheumatology clinic in a large tertiary medical center allowing greater variability in disease severity and activity and possible seasonal variations in self-report of PA. Purposefully sampling children based on age and sex, allowed for equal representation of children across these strata. While no rule exists for sample size in qualitative studies, the number of items, subjective factors, and the internal consistency of the questionnaire are important to aspects consider to reach data saturation [64]. Based on prior studies of this nature, we believe we have a robust number of children included in the trial. Adequate data-quantity and quality were met in both phases with phase 2 requiring a larger dataset to reach data saturation. The interviewer was trained by an experienced behavioral scientist, who has used cognitive interviewing for over 15 years to create and refine patient-reported outcomes. Members of the research team independently coded data and when discrepancies occurred, a normative group process was used to reach consensus.

All interviews were initiated by stating the purpose of the study. A child or adolescent may not naturally question or doubt questions provided by people in authority, to address this potential limitation, the interviewer gradually took a more informal approach to interviews to encourage the children to think and speak freely about the questions throughout the interview process. We focused this study on Swedish children as a PAQ could then potentially be added to the national JIA registry, thus these results may be limited to Swedish children with JIA. Additional limitations include the fact we did not assess the comprehensibility of these PAQ items among healthy children, nor did we test the English version of the ASCeND on native English speaking children, although this is a focus of future studies. We are currently in the process of assessing the convergent validity of the ASCeND with accelerometer estimates of PA in Swedish children with JIA and will be expanding assessment of the ASCeND to other groups of children.

## Conclusions

This study highlights the importance of assessing children’s comprehension of existing PAQ items, especially among children with JIA. We developed a new questionnaire, the ASCeND, that can be used in Swedish children with JIA to measure their PA. Assessment of PA in JIA is an area that has not been thoroughly researched. Our data indicate there are numerous issues associated with using PAQ intensity items in children, related to the concepts of strenuous, moderate and light intensity activities and that formatting of items in a survey can positively affect children’s comprehension of these items. Formatting PAQs to enable easy alignment with response options appears to reduce issues with tracking responses.

## Additional files

Additional file 1: Comparison of Content, Format and Timeframes in Published Physical Activity Measures. (DOCX 22 kb)

Additional file 2: Physical Activity Scale for Children with Different abilities (ASCenD): English version. (DOCX 85 kb)

Additional file 3: Physical Activity Scale for Children with Different abilities (ASCenD): Swedish version. (DOCX 66 kb)

## Abbreviations

ASCeND: Activity Scale for Children with Different Abilities
JIA: Juvenile idiopathic arthritis
MET: Metabolic equivalents
PA: Physical activity
PAQ: Physical activity questionnaire

## References

[1] Riddoch CJ, Bo Andersen L, Wedderkopp N, Harro M, Klasson-Heggebo L, Sardinha LB (2004). Physical activity levels and patterns of 9- and 15-yr-old European children. Med Sci Sports Exerc.

[2] Adamo KB, Langlois KA, Brett KE, Colley RC (2012). Young children and parental physical activity levels: findings from the Canadian health measures survey. Am J Prev Med.

[3] Maher CA, Williams MT, Olds T, Lane AE (2007). Physical and sedentary activity in adolescents with cerebral palsy. Dev Med Child Neurol.

[4] Buffart LM, Roebroeck ME, Rol M, Stam HJ, van den Berg-Emons RJ (2008). Transition research group south-West N. Triad of physical activity, aerobic fitness and obesity in adolescents and young adults with myelomeningocele. J Rehabil Med.

[5] Norgaard M, Herlin T (2019). Specific sports habits, leisure-time physical activity, and school-educational physical activity in children with juvenile idiopathic arthritis: patterns and barriers. Arthritis Care Res (Hoboken).

[6] Simard JF, Neovius M, Hagelberg S, Askling J (2010). Juvenile idiopathic arthritis and risk of cancer: a nationwide cohort study. Arthritis Rheum.

[7] Lelieveld OT, Armbrust W, van Leeuwen MA, Duppen N, Geertzen JH, Sauer PJ (2008). Physical activity in adolescents with juvenile idiopathic arthritis. Arthritis Rheum.

[8] Bos GJ, Lelieveld OT, Armbrust W, Sauer PJ, Geertzen JH, Dijkstra PU (2016). Physical activity in children with juvenile idiopathic arthritis compared to controls. Pediatr Rheumatol Online J..

[9] Henderson CJ, Lovell DJ, Specker BL, Campaigne BN (1995). Physical activity in children with juvenile rheumatoid arthritis: quantification and evaluation. Arthritis Care Res.

[10] Philpott J, Houghton K, Luke A (2010). Physical activity recommendations for children with specific chronic health conditions: juvenile idiopathic arthritis, hemophilia, asthma and cystic fibrosis. Paediatr Child Health.

[11] Aulie HA, Selvaag AM, Gunther A, Lilleby V, Molberg O, Hartmann A (2015). Arterial haemodynamics and coronary artery calcification in adult patients with juvenile idiopathic arthritis. Ann Rheum Dis.

[12] Wareham NJ, Rennie KL (1998). The assessment of physical activity in individuals and populations: why try to be more precise about how physical activity is assessed?. Int J Obes Relat Metab Disord.

[13] Prince SA, Adamo KB, Hamel ME, Hardt J, Connor Gorber S, Tremblay M (2008). A comparison of direct versus self-report measures for assessing physical activity in adults: a systematic review. Int J Behav Nutr Phys Act..

[14] Pedisic Z, Bauman A (2015). Accelerometer-based measures in physical activity surveillance: current practices and issues. Br J Sports Med.

[15] Kowalski K, Rhodes R, Naylor PJ, Tuokko H, MacDonald S (2012). Direct and indirect measurement of physical activity in older adults: a systematic review of the literature. Int J Behav Nutr Phys Act.

[16] Pate R, Ross R, Dowda M, Trost S, Sirard J (2003). Validation of a 3-day physical activity recall instrument in female youth. Pediatr Exerc Sci.

[17] Bonn SE, Trolle Lagerros Y, Christensen SE, Moller E, Wright A, Sjolander A (2012). Active-Q: validation of the web-based physical activity questionnaire using doubly labeled water. J Med Internet Res.

[18] Telford A, Salmon J, Jolley D, Crawford D (2004). Reliability and validity of physical activity questionnaires for children: the Children’s leisure activities study survey (CLASS). Pediatr Exerc Sci.

[19] Crocker PR, Bailey DA, Faulkner RA, Kowalski KC, McGrath R (1997). Measuring general levels of physical activity: preliminary evidence for the physical activity questionnaire for older children. Med Sci Sports Exerc.

[20] Benitez-Porres J, Lopez-Fernandez I, Raya JF, Alvarez Carnero S, Alvero-Cruz JR, Alvarez CE (2016). Reliability and validity of the PAQ-C questionnaire to assess physical activity in children. J Sch Health.

[21] Zuazagoitia A, Montoya I, Grandes G, Arietaleanizbeascoa MS, Arce V, Martinez V (2014). Reliability and validity of the 7-day physical activity recall interview in a Spanish population. Eur J Sport Sci.

[22] Booth ML, Okely AD, Chey TN, Bauman A (2002). The reliability and validity of the adolescent physical activity recall questionnaire. Med Sci Sports Exerc.

[23] Chinapaw MJ, Slootmaker SM, Schuit AJ, van Zuidam M, van Mechelen W (2009). Reliability and validity of the activity questionnaire for adults and adolescents (AQuAA). BMC Med Res Methodol.

[24] Hardy LL, Booth ML, Okely AD (2007). The reliability of the adolescent sedentary activity questionnaire (ASAQ). Prev Med.

[25] Treuth MS, Hou N, Young DR, Maynard LM (2005). Validity and reliability of the Fels physical activity questionnaire for children. Med Sci Sports Exerc.

[26] Godin G, Shephard RJ (1985). A simple method to assess exercise behavior in the community. Can J Appl Sport Sci.

[27] Bellows LL, Johnson SL, Davies PL, Anderson J, Gavin WJ, Boles RE (2013). The Colorado LEAP study: rationale and design of a study to assess the short term longitudinal effectiveness of a preschool nutrition and physical activity program. BMC Public Health.

[28] Prochaska JJ, Sallis JF, Long B (2001). A physical activity screening measure for use with adolescents in primary care. Arch Pediatr Adolesc Med.

[29] Brown TD, Holland BV (2004). Test-retest reliability of the self-assessed physical activity checklist. Percept Mot Skills.

[30] McCrorie PRW, Perez A, Ellaway A (2016). The validity of the youth physical activity questionnaire in 12-13-year-old Scottish adolescents. BMJ Open Sport Exerc Med.

[31] Singh-Grewal D, Schneiderman-Walker J, Wright V, Bar-Or O, Beyene J, Selvadurai H (2007). The effects of vigorous exercise training on physical function in children with arthritis: a randomized, controlled, single-blinded trial. Arthritis Rheum.

[32] Iversen MD, Lee B, Connell P, Andersen J, Anderson AF, Kocher MS (2010). Validity and comprehensibility of the international knee documentation committee subjective knee evaluation form in children. Scand J Med Sci Sports.

[33] Ortqvist M, Roos EM, Brostrom EW, Janarv PM, Iversen MD (2012). Development of the knee injury and osteoarthritis outcome score for children (KOOS-child): comprehensibility and content validity. Acta Orthop.

[34] Iversen MD, von Heideken J, Farmer E, Rihm J, Heyworth BE, Kocher MS (2016). Validity and comprehensibility of physical activity scales for children with sport injuries. J Pediatr Orthop.

[35] Heyworth B, Cohen L, von Heideken J, Kocher MS, Iversen MD. Validity and comprehensibility of outcome measures in children with shoulder and elbow disorders: creation of a new pediatric and adolescent shoulder and elbow survey (Pedi-ASES). J Shoulder Elb Surg. 2018. 10.1016/j.jse.2017.11.009 29307670

[36] Consolaro A, Ruperto N, Bazso A, Pistorio A, Magni-Manzoni S, Filocamo G (2009). Development and validation of a composite disease activity score for juvenile idiopathic arthritis. Arthritis Rheum.

[37] Consolaro A, Giancane G, Schiappapietra B, Davi S, Calandra S, Lanni S (2016). Clinical outcome measures in juvenile idiopathic arthritis. Pediatr Rheumatol Online J..

[38] Willis GB (2005). Cognitive interviewing : a tool for improving questionnaire design.

[39] Kocher MS, Smith JT, Iversen MD, Brustowicz K, Ogunwole O, Andersen J (2011). Reliability, validity, and responsiveness of a modified international knee documentation committee subjective knee form (Pedi-IKDC) in children with knee disorders. Am J Sports Med.

[40] Ortqvist M, Iversen MD, Janarv PM, Brostrom EW, Roos EM (2014). Psychometric properties of the knee injury and osteoarthritis outcome score for children (KOOS-child) in children with knee disorders. Br J Sports Med.

[41] Campanelli PC, Martin EA, Rothgeb JM (1991). The Use of Respondent and Interviewer Debriefing Studies as a Way to Study Response Error in Survey Data. J Royal Stat Soc Ser D (the Statistician).

[42] Tourangeau R, Rips LJ, Rasinski KA (2000). The psychology of survey response.

[43] Jobe JB, Tourangeau R, Smith AF (1993). Contributions of survey research to the understanding of memory. Appl Cogn Psychol.

[44] Tourangeau R, Jabine T, Straf M, Tanur J, Tourangeau R (1984). Cognitive sciences and survey methods. Cognitive aspects of survey methodology: building a bridge between disciplines.

[45] Holtebekk ME, Berntsen S, Rasmussen M, Jahnsen RB (2013). Physical activity and motor function in children and adolescents with neuromuscular disorders. Pediatr Phys Ther.

[46] Corder K, Ekelund U, Steele RM, Wareham NJ, Brage S (2008). Assessment of physical activity in youth. J Appl Physiol (1985).

[47] White L, Volfson Z, Faulkner G, Arbour-Nicitopoulos K (2016). Reliability and validity of physical activity instruments used in children and youth with physical disabilities: a systematic review. Pediatr Exerc Sci.

[48] Armbrust W, Bos G, Geertzen JHB, Sauer PJJ, Dijkstra PU, Lelieveld O (2017). Measuring physical activity in juvenile idiopathic arthritis: activity diary versus accelerometer. J Rheumatol.

[49] Baranowski T (1988). Validity and reliability of self report measures of physical activity: an information-processing perspective. Res Q Exerc Sport.

[50] Bailey RC, Olson J, Pepper SL, Porszasz J, Barstow TJ, Cooper DM (1995). The level and tempo of children's physical activities: an observational study. Med Sci Sports Exerc.

[51] Noland M, Danner F, DeWalt K, McFadden M, Kotchen JM (1990). The measurement of physical activity in young children. Res Q Exerc Sport.

[52] Baranowski T, de Moor C (2000). How many days was that? Intra-individual variability and physical activity assessment. Res Q Exerc Sport.

[53] Hagstromer M, Oja P, Sjostrom M (2006). The international physical activity questionnaire (IPAQ): a study of concurrent and construct validity. Public Health Nutr.

[54] Gueddari S, Amine B, Rostom S, Badri D, Mawani N, Ezzahri M (2014). Physical activity, functional ability, and disease activity in children and adolescents with juvenile idiopathic arthritis. Clin Rheumatol.

[55] Hulsegge G, Henschke N, McKay D, Chaitow J, West K, Broderick C (2015). Fundamental movement skills, physical fitness and physical activity among Australian children with juvenile idiopathic arthritis. J Paediatr Child Health.

[56] Lelieveld OT, Armbrust W, Geertzen JH, de Graaf I, van Leeuwen MA, Sauer PJ (2010). Promoting physical activity in children with juvenile idiopathic arthritis through an internet-based program: results of a pilot randomized controlled trial. Arthritis Care Res (Hoboken)..

[57] Limenis E, Grosbein HA, Feldman BM (2014). The relationship between physical activity levels and pain in children with juvenile idiopathic arthritis. J Rheumatol.

[58] Tarakci E, Yeldan I, Kaya Mutlu E, Baydogan SN, Kasapcopur O (2011). The relationship between physical activity level, anxiety, depression, and functional ability in children and adolescents with juvenile idiopathic arthritis. Clin Rheumatol.

[59] Milatz F, Klotsche J, Niewerth M, Geisemeyer N, Trauzeddel R, Weissbarth-Riedel E (2019). Participation in school sports among children and adolescents with juvenile idiopathic arthritis in the German National Paediatric Rheumatologic Database, 2000-2015: results from a prospective observational cohort study. Pediatr Rheumatol Online J.

[60] Marche TA, Briere JL, von Baeyer CL (2016). Children's forgetting of pain-related memories. J Pediatr Psychol.

[61] Ilowite NT, Walco GA, Pochaczevsky R (1992). Assessment of pain in patients with juvenile rheumatoid arthritis: relation between pain intensity and degree of joint inflammation. Ann Rheum Dis.

[62] Hagglund KJ, Schopp LM, Alberts KR, Cassidy JT, Frank RG (1995). Predicting pain among children with juvenile rheumatoid arthritis. Arthritis Care Res.

[63] Malleson PN, Oen K, Cabral DA, Petty RE, Rosenberg AM, Cheang M (2004). Predictors of pain in children with established juvenile rheumatoid arthritis. Arthritis Rheum.

[64] Blair J, Srinath K (2008). A note on sample size for behavior coding pretests. Field methods.

