# Supplementary material for: A qualitative study examining the validity and comprehensibility of physical activity items: developed and tested in children with juvenile idiopathic arthritis
Source: Pediatr Rheumatol Online J. 2019 Apr 25;17:16. doi: 10.1186/s12969-019-0317-6 (PMC6482510; doi:10.1186/s12969-019-0317-6)
Supplement: Supplementary file 1 — Comparison of Content, Format and Timeframes in Published Physical Activity Measures. (DOCX 22 kb) [file 12969_2019_317_MOESM1_ESM.docx]

**Additional file 1:** Comparison of Content, Format and Timeframes in Published Physical Activity Measures

| **Author** | **Measure** | **Timeframe and format** | **Number and Type of Items** |
| --- | --- | --- | --- |
| Pate et al.[15] | 3DPAR | Record daily PA in a timesheet. Write in clock time, identify activity by a number from sheet, and indicate intensity. | 59 PA options. Activities are numbered and categorized as follows: eating, work, afterschool/spare time; transport; sleep/bathing; school; PA/sports. |
| Bonn et al.[16] | ACTIVE-Q | Timeframe – a typical week during the past year. | Activity level in occupation using Likert scale, record hours per week at occupation. Provides lists of modes of transportation, leisure time activities, and sports activities. Subject records number of days and total time per day. Asks whether individual compete in sports and to record total sleep time. |
| Telford et al.[17] | Children’s Leisure Activities Study Survey [CLASS] | Record activities during “typical week” in a table format. Identify the frequency of the activity Mon-Fri and again on weekend. | 47 activity-based questions [45 specific, 2 open-ended] where subject provides whether they "do an activity usually" [yes/no] followed by minutes Monday-Friday and another field for the weekend. |
| Crocker et al.[18] | PAQ-A | Record activities over the last 7 days. Grid format for specific activity items, Likert response for other items. | 22 sports-specific items listed plus two open and optional questions. Items regarding what children do at lunch, frequency of sports before and after school, evenings, and weekends plus rating their activity level. Includes an item to report whether child was sick during this timeframe. |
| Benitez-Porres et al.[19] | PAQ-C | Similar to PAQ-A | Similar to PAQ-A plus asks frequency of sports participation at recess and in PE class. |
| Zuazagoitia et al.[20] | 7-Day PAR | Interview format. Subjects complete a timesheet per day for 7 days. Write in sleep hours, activities in morning, afternoon and evening by intensity level plus total minutes per day of flexibility and strengthening. | Employment, days worked per week, total hours worked. Subject records activity [no list provided] also compares PA level to PA level in past 3 months. Interview questions regarding any problems with the questionnaire, circumstances preventing PA and other questionnaire related-feedback from informant. |
| Booth et al.[21] | Adolescent Physical Activity and Recall Questionnaire [APARQ] | Table format. Child records the frequency and amount of time engaged in organized and non- organized sports at school, before and after school and on weekends during the SUMMER; stratified by season. | Child writes in the activity; no list of activities provided. Maximum of 7 activities per organized/unorganized sport per winter or summer section [maximum activities provided are 28]. |
| Chinapaw et al.[22] | Activity Questionnaire for Adults & Adolescents  AQUAA | Report days per week during the past 7 days, average time, and effort [slow, moderate, fast] through checkbox format. | Four areas of questions [commuting, activity in school, household and leisure time]. Uses closed-ended questions [16 activities] with days per week, average time per day and expected effort. Fifth area [active sports, 3 optional activities] uses open-ended activities for informant to fill in.  Each item has a "Non applicable" option. |
| Hardy et al.[23] | Adolescent Sedentary Activity  Questionnaire [ASAQ] | Table format. Report sedentary activities over a “normal school week” and on “weekend” by day and amount of time. | 11 items provided during normal school week and 12 items for the weekend. |
| Treuth et al.[24] | FELS PAQ | Items with 3-point response set [regularly to sometimes. Timeframe over the last year. | Write in options for sports played at school, sports or games outside of school, Record frequency for walking to school, biking, watching TV or playing sports, chores done at home and frequency child sweats during these activities. |
| Godin and Shepard [25] | Godin Leisure-Time Exercise Questionnaire | Item format. During a typical 7 day period, how often on average, does the child exercises for > 15 minutes during free time | Four items. Three are intensity based [mild, moderate, strenuous with examples of activities listed for each. One item regarding sweating frequency. |
| Bellows et al.[26] | LEAP II | Items with Likert responses. Categorized into 13 sections. Timeframe either not specified to indicate current activity or over coming 7 days. | 163 total items. Items reporting whether child is physically active under various conditions [agree to disagree], intention to be active over next 7 days, attitudes, beliefs, pros and cons of being active, who the child is active with, assessment of personality traits [lazy, easily bored], and perceived social support for exercise. |
| Prochaska et al.[27] | Physician-based Assessment and Counseling for Exercise [PACE+] | How often the child does PA for 60 minutes or more in a typical week & in a usual week. | 2 items with scale ranging 0-8 [0 days – 7 days] to ascertain frequency of being physically active for a total of at least 60 min per day. |
| Brown and Holland [28] | Self-administered PA checklist [SAPAC] | Participation in activities before school, during school and after school and duration of said activity ranging from none-most [1-3].  Total duration for sedentary activities.  24hour recall. | 2 items regarding PE-class duration & frequency. 2 items regarding recess duration & frequency. 21 specific activity items, 3 open-activity items with participation yes/no for before school, during & after school as well as scale of duration [none-most, 1-3]. 2 sedentary items for TV/video & video/computer games with hours & minutes before and after school. |
| McCrorie et al.[29] | Youth physical activity questionnaire [YPAQ] | How often the child does an activity on weekdays & weekends & total minutes doing the activity.  Past 7 days recall | 46 activities [PA-questions are both general PA and sedentary behaviors only asking for "free time"]. Each item child reports how often past 7 days on weekdays & weekends and total time doing it for respective weekday and weekend. |

PA = physical activity

PE = physical education class
