# Supplementary material for: A qualitative study examining the validity and comprehensibility of physical activity items: developed and tested in children with juvenile idiopathic arthritis
Source: Pediatr Rheumatol Online J. 2019 Apr 25;17:16. doi: 10.1186/s12969-019-0317-6 (PMC6482510; doi:10.1186/s12969-019-0317-6)
Supplement: Supplementary file 2 — Physical Activity Scale for Children with Different abilities (ASCenD): English version. (DOCX 85 kb) [file 12969_2019_317_MOESM2_ESM.docx]

**Physical Activity Scale for Children with Different abilities (ASCenD)**

Please read each item carefully. Answer all items as best as you can. We are collecting information on the types and amount of physical activities you have done over the PAST 7 days.

**Over the past 7 days, which of the following activities have you done, on which days per week and for how long?**

| **Walking** | | | | | | | |
| --- | --- | --- | --- | --- | --- | --- | --- |
| Time per day. | **Mon** | **Tue** | **Wed** | **Thu** | **Fri** | **Sat** | **Sun** |
| 0 min |  |  |  |  |  |  |  |
| 1-14min |  |  |  |  |  |  |  |
| 15-29 min |  |  |  |  |  |  |  |
| 30-44 min |  |  |  |  |  |  |  |
| 45-59 min |  |  |  |  |  |  |  |
| 1-2 hours |  |  |  |  |  |  |  |
| 2-4 hours |  |  |  |  |  |  |  |
| More than 4 hours |  |  |  |  |  |  |  |
| **Cycling** | | | | | | | |
| Time per day. | **Mon** | **Tue** | **Wed** | **Thu** | **Fri** | **Sat** | **Sun** |
| 0 min |  |  |  |  |  |  |  |
| 1-14min |  |  |  |  |  |  |  |
| 15-29 min |  |  |  |  |  |  |  |
| 30-44 min |  |  |  |  |  |  |  |
| 45-59 min |  |  |  |  |  |  |  |
| 1-2 hours |  |  |  |  |  |  |  |
| 2-4 hours |  |  |  |  |  |  |  |
| More than 4 hours |  |  |  |  |  |  |  |

| **By moped or scooter** | | | | | | | |
| --- | --- | --- | --- | --- | --- | --- | --- |
| Time per day. | **Mon** | **Tue** | **Wed** | **Thu** | **Fri** | **Sat** | **Sun** |
| 0 min |  |  |  |  |  |  |  |
| 1-14min |  |  |  |  |  |  |  |
| 15-29 min |  |  |  |  |  |  |  |
| 30-44 min |  |  |  |  |  |  |  |
| 45-59 min |  |  |  |  |  |  |  |
| 1-2 hours |  |  |  |  |  |  |  |
| 2-4 hours |  |  |  |  |  |  |  |
| More than 4 hours |  |  |  |  |  |  |  |
| **Riding in a vehicle (car, train, bus,** **subway or ferry** | | | | | | | |
| Time per day. | **Mon** | **Tue** | **Wed** | **Thu** | **Fri** | **Sat** | **Sun** |
| 0 min |  |  |  |  |  |  |  |
| 1-14min |  |  |  |  |  |  |  |
| 15-29 min |  |  |  |  |  |  |  |
| 30-44 min |  |  |  |  |  |  |  |
| 45-59 min |  |  |  |  |  |  |  |
| 1-2 hours |  |  |  |  |  |  |  |
| 2-4 hours |  |  |  |  |  |  |  |
| More than 4 hours |  |  |  |  |  |  |  |
| **Sat down and read a book, writing or sewing** | | | | | | | |
| Time per day. | **Mon** | **Tue** | **Wed** | **Thu** | **Fri** | **Sat** | **Sun** |
| 0 min |  |  |  |  |  |  |  |
| 1-14min |  |  |  |  |  |  |  |
| 15-29 min |  |  |  |  |  |  |  |
| 30-44 min |  |  |  |  |  |  |  |
| 45-59 min |  |  |  |  |  |  |  |
| 1-2 hours |  |  |  |  |  |  |  |
| 2-4 hours |  |  |  |  |  |  |  |
| More than 4 hours |  |  |  |  |  |  |  |
| **Watch movies, series or a show** | | | | | | | |
| Time per day. | **Mon** | **Tue** | **Wed** | **Thu** | **Fri** | **Sat** | **Sun** |
| 0 min |  |  |  |  |  |  |  |
| 1-14min |  |  |  |  |  |  |  |
| 15-29 min |  |  |  |  |  |  |  |
| 30-44 min |  |  |  |  |  |  |  |
| 45-59 min |  |  |  |  |  |  |  |
| 1-2 hours |  |  |  |  |  |  |  |
| 2-4 hours |  |  |  |  |  |  |  |
| More than 4 hours |  |  |  |  |  |  |  |
| **Played computer or TV-games** | | | | | | | |
| Time per day. | **Mon** | **Tue** | **Wed** | **Thu** | **Fri** | **Sat** | **Sun** |
| 0 min |  |  |  |  |  |  |  |
| 1-14min |  |  |  |  |  |  |  |
| 15-29 min |  |  |  |  |  |  |  |
| 30-44 min |  |  |  |  |  |  |  |
| 45-59 min |  |  |  |  |  |  |  |
| 1-2 hours |  |  |  |  |  |  |  |
| 2-4 hours |  |  |  |  |  |  |  |
| More than 4 hours |  |  |  |  |  |  |  |
| **Played a music instrument, computer-**  **Or TV-game where you are standing up or moving** | | | | | | | |
| Time per day. | **Mon** | **Tue** | **Wed** | **Thu** | **Fri** | **Sat** | **Sun** |
| 0 min |  |  |  |  |  |  |  |
| 1-14min |  |  |  |  |  |  |  |
| 15-29 min |  |  |  |  |  |  |  |
| 30-44 min |  |  |  |  |  |  |  |
| 45-59 min |  |  |  |  |  |  |  |
| 1-2 hours |  |  |  |  |  |  |  |
| 2-4 hours |  |  |  |  |  |  |  |
| More than 4 hours |  |  |  |  |  |  |  |
| **Doing household chores, cleaning**  **Laundry, taking out the trash etc.** | | | | | | | |
| Time per day. | **Mon** | **Tue** | **Wed** | **Thu** | **Fri** | **Sat** | **Sun** |
| 0 min |  |  |  |  |  |  |  |
| 1-14min |  |  |  |  |  |  |  |
| 15-29 min |  |  |  |  |  |  |  |
| 30-44 min |  |  |  |  |  |  |  |
| 45-59 min |  |  |  |  |  |  |  |
| 1-2 hours |  |  |  |  |  |  |  |
| 2-4 hours |  |  |  |  |  |  |  |
| More than 4 hours |  |  |  |  |  |  |  |
| **Shopping or other errands** | | | | | | | |
| Time per day. | **Mon** | **Tue** | **Wed** | **Thu** | **Fri** | **Sat** | **Sun** |
| 0 min |  |  |  |  |  |  |  |
| 1-14min |  |  |  |  |  |  |  |
| 15-29 min |  |  |  |  |  |  |  |
| 30-44 min |  |  |  |  |  |  |  |
| 45-59 min |  |  |  |  |  |  |  |
| 1-2 hours |  |  |  |  |  |  |  |
| 2-4 hours |  |  |  |  |  |  |  |
| More than 4 hours |  |  |  |  |  |  |  |
| **Aerobics or cardio fitness class (e.g. zumba, core, bodypump)** | | | | | | | |
| Time per day. | **Mon** | **Tue** | **Wed** | **Thu** | **Fri** | **Sat** | **Sun** |
| 0 min |  |  |  |  |  |  |  |
| 1-14min |  |  |  |  |  |  |  |
| 15-29 min |  |  |  |  |  |  |  |
| 30-44 min |  |  |  |  |  |  |  |
| 45-59 min |  |  |  |  |  |  |  |
| 1-2 hours |  |  |  |  |  |  |  |
| 2-4 hours |  |  |  |  |  |  |  |
| More than 4 hours |  |  |  |  |  |  |  |

| **Weight lifting** | | | | | | | |
| --- | --- | --- | --- | --- | --- | --- | --- |
| Time per day. | **Mon** | **Tue** | **Wed** | **Thu** | **Fri** | **Sat** | **Sun** |
| 0 min |  |  |  |  |  |  |  |
| 1-14min |  |  |  |  |  |  |  |
| 15-29 min |  |  |  |  |  |  |  |
| 30-44 min |  |  |  |  |  |  |  |
| 45-59 min |  |  |  |  |  |  |  |
| 1-2 hours |  |  |  |  |  |  |  |
| 2-4 hours |  |  |  |  |  |  |  |
| More than 4 hours |  |  |  |  |  |  |  |
| **Jogging, running or orienteering** | | | | | | | |
| Time per day. | **Mon** | **Tue** | **Wed** | **Thu** | **Fri** | **Sat** | **Sun** |
| 0 min |  |  |  |  |  |  |  |
| 1-14min |  |  |  |  |  |  |  |
| 15-29 min |  |  |  |  |  |  |  |
| 30-44 min |  |  |  |  |  |  |  |
| 45-59 min |  |  |  |  |  |  |  |
| 1-2 hours |  |  |  |  |  |  |  |
| 2-4 hours |  |  |  |  |  |  |  |
| More than 4 hours |  |  |  |  |  |  |  |
| **Athletics (e.g. high jump, long jump or three-step)** | | | | | | | |
| Time per day. | **Mon** | **Tue** | **Wed** | **Thu** | **Fri** | **Sat** | **Sun** |
| 0 min |  |  |  |  |  |  |  |
| 1-14min |  |  |  |  |  |  |  |
| 15-29 min |  |  |  |  |  |  |  |
| 30-44 min |  |  |  |  |  |  |  |
| 45-59 min |  |  |  |  |  |  |  |
| 1-2 hours |  |  |  |  |  |  |  |
| 2-4 hours |  |  |  |  |  |  |  |
| More than 4 hours |  |  |  |  |  |  |  |

| **Swimming** | | | | | | | |
| --- | --- | --- | --- | --- | --- | --- | --- |
| Time per day. | **Mon** | **Tue** | **Wed** | **Thu** | **Fri** | **Sat** | **Sun** |
| 0 min |  |  |  |  |  |  |  |
| 1-14min |  |  |  |  |  |  |  |
| 15-29 min |  |  |  |  |  |  |  |
| 30-44 min |  |  |  |  |  |  |  |
| 45-59 min |  |  |  |  |  |  |  |
| 1-2 hours |  |  |  |  |  |  |  |
| 2-4 hours |  |  |  |  |  |  |  |
| More than 4 hours |  |  |  |  |  |  |  |
| **Ball sports (e.g. soccer, basketball, volley ball or floor-ball)** | | | | | | | |
| Time per day. | **Mon** | **Tue** | **Wed** | **Thu** | **Fri** | **Sat** | **Sun** |
| 0 min |  |  |  |  |  |  |  |
| 1-14min |  |  |  |  |  |  |  |
| 15-29 min |  |  |  |  |  |  |  |
| 30-44 min |  |  |  |  |  |  |  |
| 45-59 min |  |  |  |  |  |  |  |
| 1-2 hours |  |  |  |  |  |  |  |
| 2-4 hours |  |  |  |  |  |  |  |
| More than 4 hours |  |  |  |  |  |  |  |
| **Golf** | | | | | | | |
| Time per day. | **Mon** | **Tue** | **Wed** | **Thu** | **Fri** | **Sat** | **Sun** |
| 0 min |  |  |  |  |  |  |  |
| 1-14min |  |  |  |  |  |  |  |
| 15-29 min |  |  |  |  |  |  |  |
| 30-44 min |  |  |  |  |  |  |  |
| 45-59 min |  |  |  |  |  |  |  |
| 1-2 hours |  |  |  |  |  |  |  |
| 2-4 hours |  |  |  |  |  |  |  |
| More than 4 hours |  |  |  |  |  |  |  |
| **Horseback riding** | | | | | | | |
| Time per day. | **Mon** | **Tue** | **Wed** | **Thu** | **Fri** | **Sat** | **Sun** |
| 0 min |  |  |  |  |  |  |  |
| 1-14min |  |  |  |  |  |  |  |
| 15-29 min |  |  |  |  |  |  |  |
| 30-44 min |  |  |  |  |  |  |  |
| 45-59 min |  |  |  |  |  |  |  |
| 1-2 hours |  |  |  |  |  |  |  |
| 2-4 hours |  |  |  |  |  |  |  |
| More than 4 hours |  |  |  |  |  |  |  |
| **Dance** | | | | | | | |
| Time per day. | **Mon** | **Tue** | **Wed** | **Thu** | **Fri** | **Sat** | **Sun** |
| 0 min |  |  |  |  |  |  |  |
| 1-14min |  |  |  |  |  |  |  |
| 15-29 min |  |  |  |  |  |  |  |
| 30-44 min |  |  |  |  |  |  |  |
| 45-59 min |  |  |  |  |  |  |  |
| 1-2 hours |  |  |  |  |  |  |  |
| 2-4 hours |  |  |  |  |  |  |  |
| More than 4 hours |  |  |  |  |  |  |  |
| **Dance-class or  competitive dancing** | | | | | | | |
| Time per day. | **Mon** | **Tue** | **Wed** | **Thu** | **Fri** | **Sat** | **Sun** |
| 0 min |  |  |  |  |  |  |  |
| 1-14min |  |  |  |  |  |  |  |
| 15-29 min |  |  |  |  |  |  |  |
| 30-44 min |  |  |  |  |  |  |  |
| 45-59 min |  |  |  |  |  |  |  |
| 1-2 hours |  |  |  |  |  |  |  |
| 2-4 hours |  |  |  |  |  |  |  |
| More than 4 hours |  |  |  |  |  |  |  |

| **Skating, ice-hockey** | | | | | | | |
| --- | --- | --- | --- | --- | --- | --- | --- |
| Time per day. | **Mon** | **Tue** | **Wed** | **Thu** | **Fri** | **Sat** | **Sun** |
| 0 min |  |  |  |  |  |  |  |
| 1-14min |  |  |  |  |  |  |  |
| 15-29 min |  |  |  |  |  |  |  |
| 30-44 min |  |  |  |  |  |  |  |
| 45-59 min |  |  |  |  |  |  |  |
| 1-2 hours |  |  |  |  |  |  |  |
| 2-4 hours |  |  |  |  |  |  |  |
| More than 4 hours |  |  |  |  |  |  |  |
| **Skiing (downhill or cross-country)** | | | | | | | |
| Time per day. | **Mon** | **Tue** | **Wed** | **Thu** | **Fri** | **Sat** | **Sun** |
| 0 min |  |  |  |  |  |  |  |
| 1-14min |  |  |  |  |  |  |  |
| 15-29 min |  |  |  |  |  |  |  |
| 30-44 min |  |  |  |  |  |  |  |
| 45-59 min |  |  |  |  |  |  |  |
| 1-2 hours |  |  |  |  |  |  |  |
| 2-4 hours |  |  |  |  |  |  |  |
| More than 4 hours |  |  |  |  |  |  |  |
| **Martial arts (e.g. judo or karate)** | | | | | | | |
| Time per day. | **Mon** | **Tue** | **Wed** | **Thu** | **Fri** | **Sat** | **Sun** |
| 0 min |  |  |  |  |  |  |  |
| 1-14min |  |  |  |  |  |  |  |
| 15-29 min |  |  |  |  |  |  |  |
| 30-44 min |  |  |  |  |  |  |  |
| 45-59 min |  |  |  |  |  |  |  |
| 1-2 hours |  |  |  |  |  |  |  |
| 2-4 hours |  |  |  |  |  |  |  |
| More than 4 hours |  |  |  |  |  |  |  |

| **Boxing or wrestling** | | | | | | | |
| --- | --- | --- | --- | --- | --- | --- | --- |
| Time per day. | **Mon** | **Tue** | **Wed** | **Thu** | **Fri** | **Sat** | **Sun** |
| 0 min |  |  |  |  |  |  |  |
| 1-14min |  |  |  |  |  |  |  |
| 15-29 min |  |  |  |  |  |  |  |
| 30-44 min |  |  |  |  |  |  |  |
| 45-59 min |  |  |  |  |  |  |  |
| 1-2 hours |  |  |  |  |  |  |  |
| 2-4 hours |  |  |  |  |  |  |  |
| More than 4 hours |  |  |  |  |  |  |  |
| **Tennis, badminton eller**  **Table tennis** | | | | | | | |
| Time per day. | **Mon** | **Tue** | **Wed** | **Thu** | **Fri** | **Sat** | **Sun** |
| 0 min |  |  |  |  |  |  |  |
| 1-14min |  |  |  |  |  |  |  |
| 15-29 min |  |  |  |  |  |  |  |
| 30-44 min |  |  |  |  |  |  |  |
| 45-59 min |  |  |  |  |  |  |  |
| 1-2 hours |  |  |  |  |  |  |  |
| 2-4 hours |  |  |  |  |  |  |  |
| More than 4 hours |  |  |  |  |  |  |  |
| **Squash** | | | | | | | |
| Time per day. | **Mon** | **Tue** | **Wed** | **Thu** | **Fri** | **Sat** | **Sun** |
| 0 min |  |  |  |  |  |  |  |
| 1-14min |  |  |  |  |  |  |  |
| 15-29 min |  |  |  |  |  |  |  |
| 30-44 min |  |  |  |  |  |  |  |
| 45-59 min |  |  |  |  |  |  |  |
| 1-2 hours |  |  |  |  |  |  |  |
| 2-4 hours |  |  |  |  |  |  |  |
| More than 4 hours |  |  |  |  |  |  |  |
| **Sailing, surfing, canoeingor rowing** | | | | | | | |
| Time per day. | **Mon** | **Tue** | **Wed** | **Thu** | **Fri** | **Sat** | **Sun** |
| 0 min |  |  |  |  |  |  |  |
| 1-14min |  |  |  |  |  |  |  |
| 15-29 min |  |  |  |  |  |  |  |
| 30-44 min |  |  |  |  |  |  |  |
| 45-59 min |  |  |  |  |  |  |  |
| 1-2 hours |  |  |  |  |  |  |  |
| 2-4 hours |  |  |  |  |  |  |  |
| More than 4 hours |  |  |  |  |  |  |  |
| **Motor sports (e.g. motocross)** | | | | | | | |
| Time per day. | **Mon** | **Tue** | **Wed** | **Thu** | **Fri** | **Sat** | **Sun** |
| 0 min |  |  |  |  |  |  |  |
| 1-14min |  |  |  |  |  |  |  |
| 15-29 min |  |  |  |  |  |  |  |
| 30-44 min |  |  |  |  |  |  |  |
| 45-59 min |  |  |  |  |  |  |  |
| 1-2 hours |  |  |  |  |  |  |  |
| 2-4 hours |  |  |  |  |  |  |  |
| More than 4 hours |  |  |  |  |  |  |  |
| **Rock climbing** | | | | | | | |
| Time per day. | **Mon** | **Tue** | **Wed** | **Thu** | **Fri** | **Sat** | **Sun** |
| 0 min |  |  |  |  |  |  |  |
| 1-14min |  |  |  |  |  |  |  |
| 15-29 min |  |  |  |  |  |  |  |
| 30-44 min |  |  |  |  |  |  |  |
| 45-59 min |  |  |  |  |  |  |  |
| 1-2 hours |  |  |  |  |  |  |  |
| 2-4 hours |  |  |  |  |  |  |  |
| More than 4 hours |  |  |  |  |  |  |  |

| **Yoga, tai-chi or pilates** | | | | | | | |
| --- | --- | --- | --- | --- | --- | --- | --- |
| Time per day. | **Mon** | **Tue** | **Wed** | **Thu** | **Fri** | **Sat** | **Sun** |
| 0 min |  |  |  |  |  |  |  |
| 1-14min |  |  |  |  |  |  |  |
| 15-29 min |  |  |  |  |  |  |  |
| 30-44 min |  |  |  |  |  |  |  |
| 45-59 min |  |  |  |  |  |  |  |
| 1-2 hours |  |  |  |  |  |  |  |
| 2-4 hours |  |  |  |  |  |  |  |
| More than 4 hours |  |  |  |  |  |  |  |
| **Mountain-bike or  biking in demanding terrain** | | | | | | | |
| Time per day. | **Mon** | **Tue** | **Wed** | **Thu** | **Fri** | **Sat** | **Sun** |
| 0 min |  |  |  |  |  |  |  |
| 1-14min |  |  |  |  |  |  |  |
| 15-29 min |  |  |  |  |  |  |  |
| 30-44 min |  |  |  |  |  |  |  |
| 45-59 min |  |  |  |  |  |  |  |
| 1-2 hours |  |  |  |  |  |  |  |
| 2-4 hours |  |  |  |  |  |  |  |
| More than 4 hours |  |  |  |  |  |  |  |

**Physical activity in children (play, exercise, sports)**

**Instructions:** In this question, we are interested in knowing how physically exhausting your day is.
Please answer all the questions by placing an X in the item that describes how much physical activity you have done on average **per day** during the **past 7 days**.

|  | 0 minutes | Less than 30 minutes | 30 minutes to an hour | 1-1.5 hours | 1.5-2 hours | 2-3  hours | 3-6  hours | 6-9  hours | 9-12  hours | More than 12 hours |
| --- | --- | --- | --- | --- | --- | --- | --- | --- | --- | --- |
| **Heavily exhausting** physical activity |  |  |  |  |  |  |  |  |  |  |
| **Moderately exhausting** physical activity |  |  |  |  |  |  |  |  |  |  |
| **Not exhausting** physical activity |  |  |  |  |  |  |  |  |  |  |

How much time have you spent sitting or laying still (both at day and night) on average **per day** over **the past 7 days?**

|  | 0 minutes | Less than 30 minutes. | 30 minutes to an hour. | 1-1.5 hours | 1.5-2 Hours | 2-3  hours | 3-6  hours | 6-9  hours | 9-12  hours | More than 12 hours |
| --- | --- | --- | --- | --- | --- | --- | --- | --- | --- | --- |
| How much have you **slept** (both at day and night)? |  |  |  |  |  |  |  |  |  |  |
| How much have you been **laying** down?  ***Not sleeping*** |  |  |  |  |  |  |  |  |  |  |
| How much have you been **sitting?** |  |  |  |  |  |  |  |  |  |  |

**G1. Compared to others your age, how would you describe your activity level?**

1. I am **less** physically active than they are.
2. I am **as** physically active as they are.
3. I am **a little bit more** physically active than they are.
4. I am **a lot more** physically active than they are.

**G2. Have you been sick the last 7-days?**

1. Yes
2. No

**Thank you for taking the time to answer all of the questions!**
