# Supplementary material for: A qualitative study examining the validity and comprehensibility of physical activity items: developed and tested in children with juvenile idiopathic arthritis
Source: Pediatr Rheumatol Online J. 2019 Apr 25;17:16. doi: 10.1186/s12969-019-0317-6 (PMC6482510; doi:10.1186/s12969-019-0317-6)
Supplement: Supplementary file 3 — Physical Activity Scale for Children with Different abilities (ASCenD): Swedish version. (DOCX 66 kb) [file 12969_2019_317_MOESM3_ESM.docx]

**Physical Activity Scale for Children with Different abilities (ASCenD)**

Svara på varje fråga genom att kryssa för det alternativ du tycker är bäst (endast ett alternativ per fråga). Vi samlar information om de typer och mängden fysisk aktivitet du gör.

**Under de senaste 7 dagarna, vilka av följande aktiviteter har du gjort, antalet dagar per vecka och för hur länge?**

| **Gått** | | | | | | | |
| --- | --- | --- | --- | --- | --- | --- | --- |
| Tid per dag. | **Mån** | **Tis** | **Ons** | **Tors** | **Fre** | **Lör** | **Sön** |
| 0 min |  |  |  |  |  |  |  |
| 1-14min |  |  |  |  |  |  |  |
| 15-29 min |  |  |  |  |  |  |  |
| 30-44 min |  |  |  |  |  |  |  |
| 45-59 min |  |  |  |  |  |  |  |
| 1-2 timmar |  |  |  |  |  |  |  |
| 2-4 timmar |  |  |  |  |  |  |  |
| Mer än 4 timmar |  |  |  |  |  |  |  |
| **Cyklat** | | | | | | | |
| Tid per dag. | **Mån** | **Tis** | **Ons** | **Tors** | **Fre** | **Lör** | **Sön** |
| 0 min |  |  |  |  |  |  |  |
| 1-14min |  |  |  |  |  |  |  |
| 15-29 min |  |  |  |  |  |  |  |
| 30-44 min |  |  |  |  |  |  |  |
| 45-59 min |  |  |  |  |  |  |  |
| 1-2 timmar |  |  |  |  |  |  |  |
| 2-4 timmar |  |  |  |  |  |  |  |
| Mer än 4 timmar |  |  |  |  |  |  |  |

| **Åkt moped eller skoter** | | | | | | | |
| --- | --- | --- | --- | --- | --- | --- | --- |
| Tid per dag. | **Mån** | **Tis** | **Ons** | **Tors** | **Fre** | **Lör** | **Sön** |
| 0 min |  |  |  |  |  |  |  |
| 1-14min |  |  |  |  |  |  |  |
| 15-29 min |  |  |  |  |  |  |  |
| 30-44 min |  |  |  |  |  |  |  |
| 45-59 min |  |  |  |  |  |  |  |
| 1-2 timmar |  |  |  |  |  |  |  |
| 2-4 timmar |  |  |  |  |  |  |  |
| Mer än 4 timmar |  |  |  |  |  |  |  |
| **Åkt andra fordon (bil, tåg, buss,**  **tunnelbana eller färja)** | | | | | | | |
| Tid per dag. | **Mån** | **Tis** | **Ons** | **Tors** | **Fre** | **Lör** | **Sön** |
| 0 min |  |  |  |  |  |  |  |
| 1-14min |  |  |  |  |  |  |  |
| 15-29 min |  |  |  |  |  |  |  |
| 30-44 min |  |  |  |  |  |  |  |
| 45-59 min |  |  |  |  |  |  |  |
| 1-2 timmar |  |  |  |  |  |  |  |
| 2-4 timmar |  |  |  |  |  |  |  |
| Mer än 4 timmar |  |  |  |  |  |  |  |
| **Suttit ned och läst en bok, skrivit eller stickat** | | | | | | | |
| Tid per dag. | **Mån** | **Tis** | **Ons** | **Tors** | **Fre** | **Lör** | **Sön** |
| 0 min |  |  |  |  |  |  |  |
| 1-14min |  |  |  |  |  |  |  |
| 15-29 min |  |  |  |  |  |  |  |
| 30-44 min |  |  |  |  |  |  |  |
| 45-59 min |  |  |  |  |  |  |  |
| 1-2 timmar |  |  |  |  |  |  |  |
| 2-4 timmar |  |  |  |  |  |  |  |
| Mer än 4 timmar |  |  |  |  |  |  |  |
| **Tittat på film, serier eller program** | | | | | | | |
| Tid per dag. | **Mån** | **Tis** | **Ons** | **Tors** | **Fre** | **Lör** | **Sön** |
| 0 min |  |  |  |  |  |  |  |
| 1-14min |  |  |  |  |  |  |  |
| 15-29 min |  |  |  |  |  |  |  |
| 30-44 min |  |  |  |  |  |  |  |
| 45-59 min |  |  |  |  |  |  |  |
| 1-2 timmar |  |  |  |  |  |  |  |
| 2-4 timmar |  |  |  |  |  |  |  |
| Mer än 4 timmar |  |  |  |  |  |  |  |
| **Spelat dator eller TV-spel** | | | | | | | |
| Tid per dag. | **Mån** | **Tis** | **Ons** | **Tors** | **Fre** | **Lör** | **Sön** |
| 0 min |  |  |  |  |  |  |  |
| 1-14min |  |  |  |  |  |  |  |
| 15-29 min |  |  |  |  |  |  |  |
| 30-44 min |  |  |  |  |  |  |  |
| 45-59 min |  |  |  |  |  |  |  |
| 1-2 timmar |  |  |  |  |  |  |  |
| 2-4 timmar |  |  |  |  |  |  |  |
| Mer än 4 timmar |  |  |  |  |  |  |  |
| **Spelat musikinstrument, dator-**  **eller TV-spel där du står upp eller rör på dig** | | | | | | | |
| Tid per dag. | **Mån** | **Tis** | **Ons** | **Tors** | **Fre** | **Lör** | **Sön** |
| 0 min |  |  |  |  |  |  |  |
| 1-14min |  |  |  |  |  |  |  |
| 15-29 min |  |  |  |  |  |  |  |
| 30-44 min |  |  |  |  |  |  |  |
| 45-59 min |  |  |  |  |  |  |  |
| 1-2 timmar |  |  |  |  |  |  |  |
| 2-4 timmar |  |  |  |  |  |  |  |
| Mer än 4 timmar |  |  |  |  |  |  |  |
| **Gjort hushållssysslor (Tex städning,**  **tvättat eller tagit ut sopor)** | | | | | | | |
| Tid per dag. | **Mån** | **Tis** | **Ons** | **Tors** | **Fre** | **Lör** | **Sön** |
| 0 min |  |  |  |  |  |  |  |
| 1-14min |  |  |  |  |  |  |  |
| 15-29 min |  |  |  |  |  |  |  |
| 30-44 min |  |  |  |  |  |  |  |
| 45-59 min |  |  |  |  |  |  |  |
| 1-2 timmar |  |  |  |  |  |  |  |
| 2-4 timmar |  |  |  |  |  |  |  |
| Mer än 4 timmar |  |  |  |  |  |  |  |
| **Shopping eller gå och handla** | | | | | | | |
| Tid per dag. | **Mån** | **Tis** | **Ons** | **Tors** | **Fre** | **Lör** | **Sön** |
| 0 min |  |  |  |  |  |  |  |
| 1-14min |  |  |  |  |  |  |  |
| 15-29 min |  |  |  |  |  |  |  |
| 30-44 min |  |  |  |  |  |  |  |
| 45-59 min |  |  |  |  |  |  |  |
| 1-2 timmar |  |  |  |  |  |  |  |
| 2-4 timmar |  |  |  |  |  |  |  |
| Mer än 4 timmar |  |  |  |  |  |  |  |
| **Konditionsträningspass (t.ex.zumba,**  **core, bodypump)** | | | | | | | |
| Tid per dag. | **Mån** | **Tis** | **Ons** | **Tors** | **Fre** | **Lör** | **Sön** |
| 0 min |  |  |  |  |  |  |  |
| 1-14min |  |  |  |  |  |  |  |
| 15-29 min |  |  |  |  |  |  |  |
| 30-44 min |  |  |  |  |  |  |  |
| 45-59 min |  |  |  |  |  |  |  |
| 1-2 timmar |  |  |  |  |  |  |  |
| 2-4 timmar |  |  |  |  |  |  |  |
| Mer än 4 timmar |  |  |  |  |  |  |  |

| **Styrketräning** | | | | | | | |
| --- | --- | --- | --- | --- | --- | --- | --- |
| Tid per dag. | **Mån** | **Tis** | **Ons** | **Tors** | **Fre** | **Lör** | **Sön** |
| 0 min |  |  |  |  |  |  |  |
| 1-14min |  |  |  |  |  |  |  |
| 15-29 min |  |  |  |  |  |  |  |
| 30-44 min |  |  |  |  |  |  |  |
| 45-59 min |  |  |  |  |  |  |  |
| 1-2 timmar |  |  |  |  |  |  |  |
| 2-4 timmar |  |  |  |  |  |  |  |
| Mer än 4 timmar |  |  |  |  |  |  |  |
| **Joggning, löpning eller orientering** | | | | | | | |
| Tid per dag. | **Mån** | **Tis** | **Ons** | **Tors** | **Fre** | **Lör** | **Sön** |
| 0 min |  |  |  |  |  |  |  |
| 1-14min |  |  |  |  |  |  |  |
| 15-29 min |  |  |  |  |  |  |  |
| 30-44 min |  |  |  |  |  |  |  |
| 45-59 min |  |  |  |  |  |  |  |
| 1-2 timmar |  |  |  |  |  |  |  |
| 2-4 timmar |  |  |  |  |  |  |  |
| Mer än 4 timmar |  |  |  |  |  |  |  |
| **Friidrott (t.ex. höjdhopp, längdhopp**  **eller tre-steg** | | | | | | | |
| Tid per dag. | **Mån** | **Tis** | **Ons** | **Tors** | **Fre** | **Lör** | **Sön** |
| 0 min |  |  |  |  |  |  |  |
| 1-14min |  |  |  |  |  |  |  |
| 15-29 min |  |  |  |  |  |  |  |
| 30-44 min |  |  |  |  |  |  |  |
| 45-59 min |  |  |  |  |  |  |  |
| 1-2 timmar |  |  |  |  |  |  |  |
| 2-4 timmar |  |  |  |  |  |  |  |
| Mer än 4 timmar |  |  |  |  |  |  |  |

| **Simning** | | | | | | | |
| --- | --- | --- | --- | --- | --- | --- | --- |
| Tid per dag. | **Mån** | **Tis** | **Ons** | **Tors** | **Fre** | **Lör** | **Sön** |
| 0 min |  |  |  |  |  |  |  |
| 1-14min |  |  |  |  |  |  |  |
| 15-29 min |  |  |  |  |  |  |  |
| 30-44 min |  |  |  |  |  |  |  |
| 45-59 min |  |  |  |  |  |  |  |
| 1-2 timmar |  |  |  |  |  |  |  |
| 2-4 timmar |  |  |  |  |  |  |  |
| Mer än 4 timmar |  |  |  |  |  |  |  |
| **Bollsporter (t.ex. fotboll, basket,**  **Volleyboll eller innebandy)** | | | | | | | |
| Tid per dag. | **Mån** | **Tis** | **Ons** | **Tors** | **Fre** | **Lör** | **Sön** |
| 0 min |  |  |  |  |  |  |  |
| 1-14min |  |  |  |  |  |  |  |
| 15-29 min |  |  |  |  |  |  |  |
| 30-44 min |  |  |  |  |  |  |  |
| 45-59 min |  |  |  |  |  |  |  |
| 1-2 timmar |  |  |  |  |  |  |  |
| 2-4 timmar |  |  |  |  |  |  |  |
| Mer än 4 timmar |  |  |  |  |  |  |  |
| **Golf** | | | | | | | |
| Tid per dag. | **Mån** | **Tis** | **Ons** | **Tors** | **Fre** | **Lör** | **Sön** |
| 0 min |  |  |  |  |  |  |  |
| 1-14min |  |  |  |  |  |  |  |
| 15-29 min |  |  |  |  |  |  |  |
| 30-44 min |  |  |  |  |  |  |  |
| 45-59 min |  |  |  |  |  |  |  |
| 1-2 timmar |  |  |  |  |  |  |  |
| 2-4 timmar |  |  |  |  |  |  |  |
| Mer än 4 timmar |  |  |  |  |  |  |  |
| **Ridning** | | | | | | | |
| Tid per dag. | **Mån** | **Tis** | **Ons** | **Tors** | **Fre** | **Lör** | **Sön** |
| 0 min |  |  |  |  |  |  |  |
| 1-14min |  |  |  |  |  |  |  |
| 15-29 min |  |  |  |  |  |  |  |
| 30-44 min |  |  |  |  |  |  |  |
| 45-59 min |  |  |  |  |  |  |  |
| 1-2 timmar |  |  |  |  |  |  |  |
| 2-4 timmar |  |  |  |  |  |  |  |
| Mer än 4 timmar |  |  |  |  |  |  |  |
| **Dans** | | | | | | | |
| Tid per dag. | **Mån** | **Tis** | **Ons** | **Tors** | **Fre** | **Lör** | **Sön** |
| 0 min |  |  |  |  |  |  |  |
| 1-14min |  |  |  |  |  |  |  |
| 15-29 min |  |  |  |  |  |  |  |
| 30-44 min |  |  |  |  |  |  |  |
| 45-59 min |  |  |  |  |  |  |  |
| 1-2 timmar |  |  |  |  |  |  |  |
| 2-4 timmar |  |  |  |  |  |  |  |
| Mer än 4 timmar |  |  |  |  |  |  |  |
| **Danslektion eller**  **tävlingsdans** | | | | | | | |
| Tid per dag. | **Mån** | **Tis** | **Ons** | **Tors** | **Fre** | **Lör** | **Sön** |
| 0 min |  |  |  |  |  |  |  |
| 1-14min |  |  |  |  |  |  |  |
| 15-29 min |  |  |  |  |  |  |  |
| 30-44 min |  |  |  |  |  |  |  |
| 45-59 min |  |  |  |  |  |  |  |
| 1-2 timmar |  |  |  |  |  |  |  |
| 2-4 timmar |  |  |  |  |  |  |  |
| Mer än 4 timmar |  |  |  |  |  |  |  |

| **Skridskoåkning, ishockey eller**  **annan issport** | | | | | | | |
| --- | --- | --- | --- | --- | --- | --- | --- |
| Tid per dag. | **Mån** | **Tis** | **Ons** | **Tors** | **Fre** | **Lör** | **Sön** |
| 0 min |  |  |  |  |  |  |  |
| 1-14min |  |  |  |  |  |  |  |
| 15-29 min |  |  |  |  |  |  |  |
| 30-44 min |  |  |  |  |  |  |  |
| 45-59 min |  |  |  |  |  |  |  |
| 1-2 timmar |  |  |  |  |  |  |  |
| 2-4 timmar |  |  |  |  |  |  |  |
| Mer än 4 timmar |  |  |  |  |  |  |  |
| **Skidåkning (utför eller**  **längd)** | | | | | | | |
| Tid per dag. | **Mån** | **Tis** | **Ons** | **Tors** | **Fre** | **Lör** | **Sön** |
| 0 min |  |  |  |  |  |  |  |
| 1-14min |  |  |  |  |  |  |  |
| 15-29 min |  |  |  |  |  |  |  |
| 30-44 min |  |  |  |  |  |  |  |
| 45-59 min |  |  |  |  |  |  |  |
| 1-2 timmar |  |  |  |  |  |  |  |
| 2-4 timmar |  |  |  |  |  |  |  |
| Mer än 4 timmar |  |  |  |  |  |  |  |
| **Kampsport (t.ex. judo eller**  **kickboxning)** | | | | | | | |
| Tid per dag. | **Mån** | **Tis** | **Ons** | **Tors** | **Fre** | **Lör** | **Sön** |
| 0 min |  |  |  |  |  |  |  |
| 1-14min |  |  |  |  |  |  |  |
| 15-29 min |  |  |  |  |  |  |  |
| 30-44 min |  |  |  |  |  |  |  |
| 45-59 min |  |  |  |  |  |  |  |
| 1-2 timmar |  |  |  |  |  |  |  |
| 2-4 timmar |  |  |  |  |  |  |  |
| Mer än 4 timmar |  |  |  |  |  |  |  |

| **Boxning eller brottning** | | | | | | | |
| --- | --- | --- | --- | --- | --- | --- | --- |
| Tid per dag. | **Mån** | **Tis** | **Ons** | **Tors** | **Fre** | **Lör** | **Sön** |
| 0 min |  |  |  |  |  |  |  |
| 1-14min |  |  |  |  |  |  |  |
| 15-29 min |  |  |  |  |  |  |  |
| 30-44 min |  |  |  |  |  |  |  |
| 45-59 min |  |  |  |  |  |  |  |
| 1-2 timmar |  |  |  |  |  |  |  |
| 2-4 timmar |  |  |  |  |  |  |  |
| Mer än 4 timmar |  |  |  |  |  |  |  |
| **Tennis, badminton eller**  **bordtennis** | | | | | | | |
| Tid per dag. | **Mån** | **Tis** | **Ons** | **Tors** | **Fre** | **Lör** | **Sön** |
| 0 min |  |  |  |  |  |  |  |
| 1-14min |  |  |  |  |  |  |  |
| 15-29 min |  |  |  |  |  |  |  |
| 30-44 min |  |  |  |  |  |  |  |
| 45-59 min |  |  |  |  |  |  |  |
| 1-2 timmar |  |  |  |  |  |  |  |
| 2-4 timmar |  |  |  |  |  |  |  |
| Mer än 4 timmar |  |  |  |  |  |  |  |
| **Squash** | | | | | | | |
| Tid per dag. | **Mån** | **Tis** | **Ons** | **Tors** | **Fre** | **Lör** | **Sön** |
| 0 min |  |  |  |  |  |  |  |
| 1-14min |  |  |  |  |  |  |  |
| 15-29 min |  |  |  |  |  |  |  |
| 30-44 min |  |  |  |  |  |  |  |
| 45-59 min |  |  |  |  |  |  |  |
| 1-2 timmar |  |  |  |  |  |  |  |
| 2-4 timmar |  |  |  |  |  |  |  |
| Mer än 4 timmar |  |  |  |  |  |  |  |
| **Segling, surfing, kanot eller rodd** | | | | | | | |
| Tid per dag. | **Mån** | **Tis** | **Ons** | **Tors** | **Fre** | **Lör** | **Sön** |
| 0 min |  |  |  |  |  |  |  |
| 1-14min |  |  |  |  |  |  |  |
| 15-29 min |  |  |  |  |  |  |  |
| 30-44 min |  |  |  |  |  |  |  |
| 45-59 min |  |  |  |  |  |  |  |
| 1-2 timmar |  |  |  |  |  |  |  |
| 2-4 timmar |  |  |  |  |  |  |  |
| Mer än 4 timmar |  |  |  |  |  |  |  |
| **Motorsport (t.ex. cross)** | | | | | | | |
| Tid per dag. | **Mån** | **Tis** | **Ons** | **Tors** | **Fre** | **Lör** | **Sön** |
| 0 min |  |  |  |  |  |  |  |
| 1-14min |  |  |  |  |  |  |  |
| 15-29 min |  |  |  |  |  |  |  |
| 30-44 min |  |  |  |  |  |  |  |
| 45-59 min |  |  |  |  |  |  |  |
| 1-2 timmar |  |  |  |  |  |  |  |
| 2-4 timmar |  |  |  |  |  |  |  |
| Mer än 4 timmar |  |  |  |  |  |  |  |
| **Bergsklättring** | | | | | | | |
| Tid per dag. | **Mån** | **Tis** | **Ons** | **Tors** | **Fre** | **Lör** | **Sön** |
| 0 min |  |  |  |  |  |  |  |
| 1-14min |  |  |  |  |  |  |  |
| 15-29 min |  |  |  |  |  |  |  |
| 30-44 min |  |  |  |  |  |  |  |
| 45-59 min |  |  |  |  |  |  |  |
| 1-2 timmar |  |  |  |  |  |  |  |
| 2-4 timmar |  |  |  |  |  |  |  |
| Mer än 4 timmar |  |  |  |  |  |  |  |

| **Yoga, tai-chi eller pilates** | | | | | | | |
| --- | --- | --- | --- | --- | --- | --- | --- |
| Tid per dag. | **Mån** | **Tis** | **Ons** | **Tors** | **Fre** | **Lör** | **Sön** |
| 0 min |  |  |  |  |  |  |  |
| 1-14min |  |  |  |  |  |  |  |
| 15-29 min |  |  |  |  |  |  |  |
| 30-44 min |  |  |  |  |  |  |  |
| 45-59 min |  |  |  |  |  |  |  |
| 1-2 timmar |  |  |  |  |  |  |  |
| 2-4 timmar |  |  |  |  |  |  |  |
| Mer än 4 timmar |  |  |  |  |  |  |  |
| **Mountain-bike eller cykla i krävande terräng** | | | | | | | |
| Tid per dag. | **Mån** | **Tis** | **Ons** | **Tors** | **Fre** | **Lör** | **Sön** |
| 0 min |  |  |  |  |  |  |  |
| 1-14min |  |  |  |  |  |  |  |
| 15-29 min |  |  |  |  |  |  |  |
| 30-44 min |  |  |  |  |  |  |  |
| 45-59 min |  |  |  |  |  |  |  |
| 1-2 timmar |  |  |  |  |  |  |  |
| 2-4 timmar |  |  |  |  |  |  |  |
| Mer än 4 timmar |  |  |  |  |  |  |  |

**Fysisk aktivitet hos barn (lek, idrott, sport)**

**Instruktion:** I denna fråga är vi intresserade av att veta hur fysiskt ansträngande din vardag är.

Vänligen svara på alla frågor genom att kryssa i de alternativ som **bäst** beskriver hur mycket fysisk aktivitet som du har utfört i genomsnitt **per dag** under de **senaste 7 dagarna.**

|  | 0 minuter | Mindre än 30 minuter | 30 minuter till en timme | 1-1.5 timmar | 1.5-2 timmar | 2-3  timmar | 3-6  timmar | 6-9 timmar | 9-12 timmar | Mer än 12 timmar |
| --- | --- | --- | --- | --- | --- | --- | --- | --- | --- | --- |
| **Kraftigt ansträngande** fysiskt aktivitet |  |  |  |  |  |  |  |  |  |  |
| **Lätt ansträngande**  fysiskt aktivitet |  |  |  |  |  |  |  |  |  |  |
| **Inte ansträngande**  fysiskt aktivitet |  |  |  |  |  |  |  |  |  |  |

Hur mycket tid har du suttit eller legat stilla (både på dagen och natten) i genomsnitt **per dag** under de **senaste 7 dagarna?**

|  | 0 minuter | Mindre än 30 minuter | 30 minuter till en timme | 1-1.5 timmar | 1.5-2 timmar | 2-3  timmar | 3-6  timmar | 6-9 timmar | 9-12 timmar | Mer än 12 timmar |
| --- | --- | --- | --- | --- | --- | --- | --- | --- | --- | --- |
| Hur mycket har du **sovit** (både på natten och dagen)**?** |  |  |  |  |  |  |  |  |  |  |
| Hur mycket har du **legat** ned och vilat  ***Ej sömn*** |  |  |  |  |  |  |  |  |  |  |
| Hur mycket har du **suttit?** |  |  |  |  |  |  |  |  |  |  |

**G1. Jämfört med andra i din ålder, hur skulle du beskriva din aktivitetsnivå?**

1. Jag är **mindre** fysiskt aktiv än dem
2. Jag är **lika** fysiskt aktiv som dem
3. Jag är **lite mer** fysiskt aktiv än dem
4. Jag är **mycket mer** fysiskt aktiv än dem

**G2. Har du varit sjuk de senaste 7 dagarna?**

1. Ja
2. Nej

**Tack för att Du tagit dig tid att besvara samtliga frågor!**
